# *In Vitro* Activity of Cefepime/AAI101 and Comparators against Cefepime Non-susceptible *Enterobacteriaceae*

**DOI:** 10.3390/pathogens4030620

**Published:** 2015-08-18

**Authors:** Jared L. Crandon, David P. Nicolau

**Affiliations:** 1 Center for Anti-Infective Research and Development, Hartford Hospital, 80 Seymour Street, Hartford, CT 06102, USA; E-Mail: jared.crandon@hhchealth.org; 2 Division of Infectious Diseases, Hartford Hospital, Hartford, CT 06102, USA

**Keywords:** *Escherichia coli*, *Klebsiella pneumonia*, ESBL, carbapenemase, β-lactamase inhibitor

## Abstract

We evaluated the *in vitro* potency of cefepime combined with AAI101, a novel extended-spectrum β-lactamase inhibitor, against a population of clinical *Escherichia coli* and *Klebsiella pneumoniae* collected from USA hospitals. Of the 223 cefepime non-susceptible isolates, 95% were ceftazidime non-susceptible, 49% ertapenem non-susceptible, 57% piperacillin/tazobactam non-susceptible, 90% were multidrug-resistant (resistant to ≥3 drug classes), 22% produced carbapenemases, and 67% produced ESBLs. Addition of AAI101 restored the activity of cefepime such that the MIC_50_ was reduced from >64 mg/L for cefepime to 0.13 mg/L for cefepime/AAI101, supporting its continued development treatment for infections caused by these organisms.

## 1. Introduction

The increasing prevalence of resistance among Gram-negative pathogens has resulted in tremendous challenges for clinicians across the globe. Secondary to the pathogenicity of *Enterobacteriaceae* across diseases states, considerable attention has been given to these once highly susceptible organisms. This is particularly true for those organisms that are resistant to our most potent β-lactams, such as late-generation cephalosporins and carbapenems [1]. While non-enzymatic resistance mechanisms exist (*i.e.*, porin mutations, efflux pump overexpression), the most common cause of β-lactam resistance in *Enterobacteriaceae* is production of β-lactamases [2]. Capitalizing on this common mechanism of resistance, a combination of cefepime, a widely-used cephalosporin, and AAI101, a novel β-lactamase inhibitor with activity against extended-spectrum β-lactamases (ESBL), as well as, some class A and class D carbapenemases, is in early clinical development [3,4]. While previous studies have focused on smaller collections of genotypically characterized isolates, many of which were derived in the laboratory, this study was designed to understand the potency of this new combination against a larger, more clinically focused, yet challenging distribution [3,4]. Namely, we evaluated the *in vitro* activity of cefepime/AAI101 against a panel of highly resistant clinical isolates of *Escherichia coli* and *Klebsiella pneumoniae* collected during 2013–2014 from hospitals across the USA, and compared its activity with those of currently available therapies.

## 2. Results

**Figure 1:**
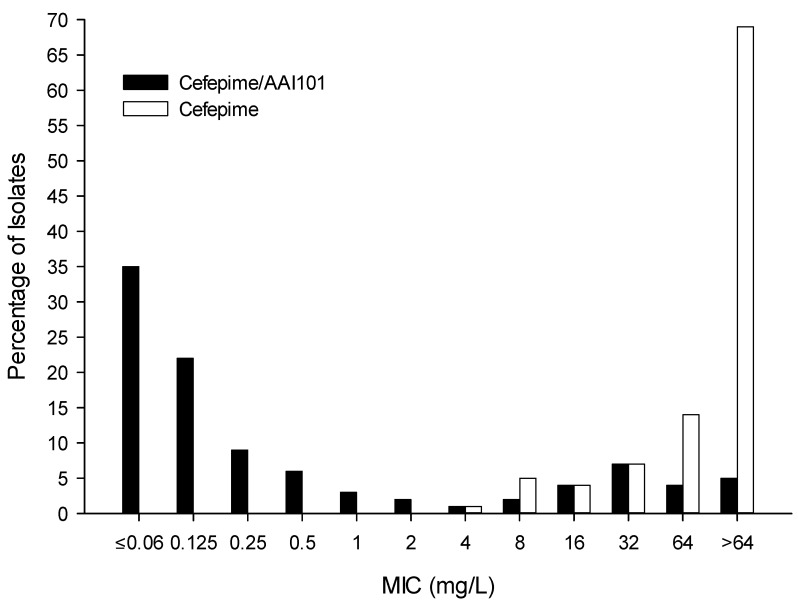
MIC distributions for cefepime and cefepime/AAI101 against 223 recent *Enterobacteriaceae* clinical isolates.

Of the 223 strains evaluated, 165 (74%) were collected from patients residing in the general ward, while the remaining 58 (26%) were from ICU patients. The majority of strains were isolated from the blood (42%), followed by wounds (21%) and the lower respiratory tract (17%). 200 (90%) strains were multidrug-resistant, 150 (67%) were confirmed ESBL producers, and 50 (22.4%) produced carbapenemases. Overall, all agents had greater activity against *E. coli* than against *K. pneumoniae*. The percent susceptibility, MIC_50_, MIC_90_, and ranges of MIC values for cefepime/AAI101 and comparators are displayed in Table 1. The cefepime/AAI101 activity against specific resistance phenotypes, including ESBL and carbapenemase producers are shown in Table 2. To highlight the ability of AAI101 to restore the potency of cefepime, Figure 1 shows the MIC distributions of cefepime and cefepime/AAI101 against the full complement of isolates.

**Table 1:** MIC profile of cefepime/AAI101 and comparator agents against the selected *Enterobacteriaceae* isolates (*n* = 223).

Antimicrobial Agent	% Susceptible	MIC_50_ (mg/L)	MIC_90_ (mg/L)	Range (mg/L)
Cefepime/AAI101	ND	0.125	64	≤0.06 to >64
Cefepime	0 *	>64	>64	4 to >64
Ceftazidime	5	>64	>64	0.5 to >64
Ciprofloxacin	9	>16	>16	≤0.015 to >16
Ertapenem	51	0.5	64	≤0.015 to >16
Meropenem	70	0.125	64	≤0.06 to >64
Piperacillin/tazobactam	43	32	>256	1 to >256
Tobramycin	40	16	64	≤0.06 to >64

ND, not defined; * 7% susceptible dose-dependent.

**Table 2:** MIC profile of cefepime/AAI101 against *Enterobacteriaceae* isolates exhibiting various resistance phenotypes.

Antimicrobial Agent	MIC_50_ (mg/L)	MIC_90_ (mg/L)	Range (mg/L)
Cefepime Resistant (*n* = 208)	0.125	64	≤0.06 to >64
Ciprofloxacin Resistant (*n* = 210)	0.125	64	≤0.06 to >64
Ertapenem Resistant (*n* = 110)	1	>64	≤0.06 to >64
Piperacillin/tazobactam Resistant (*n* = 127)	1	>64	≤0.06 to >64
Multi-drug Resistant (*n* = 200)	0.125	64	≤0.06 to >64
ESBL Producers (*n* = 150)	0.125	0.5	≤0.06 to >64
Carbapenemase Producers (*n* = 50)	32	>256	≤0.06 to >64

## 3. Discussion

Against this challenging population of *Enterobacteriaceae*, cefepime/AAI101 was quite potent based on MIC_50_ and MIC_90_ values. Although a breakpoint has not yet been established for cefepime/AAI101, if one considers its MIC distribution in the context of current cefepime breakpoints, susceptibility in this population of isolates would be greater than that of all other agents evaluated. Namely, application of the susceptible-dose dependent (SDD) breakpoint for a 2 g q8h dose of cefepime, as recommended for patients with serious infections, (*i.e.*, ≤8 mg/L) [5] to cefepime/AAI101, would result in 80% susceptibility of the strains examined in this study. While clinical pharmacokinetic and pharmacodynamic data are required to validate this assumption, experience with other β-lactam/β-lactamase inhibitor combinations suggests that when the β-lactamase inhibitor content is sufficient to restore activity, the pharmacodynamics of the β-lactam antibiotic partner prevail [6,7,8,9].

Given the number of isolates evaluated, we were unable to genetically verify β-lactamase content of the studied strains. However, through the use of phenotypic methodologies we could identify those strains that produced ESBLs or carbapenemases. As noted in Table 2, cefepime/AAI101 was quite potent against ESBL producing strains and retained activity against a proportion of carbapenemase producers. These findings are similar to previous studies of cefepime/AAI101 against small numbers of genotypically described strains that highlighted AAI101’s ability to protect cefepime against hydrolysis by ESBLs, such as TEM-type, SHV-type, CTX-M-type, and AmpC, as well as some carbapenemases, including OXA-48s and KPCs [3,4]. Of course, further studies evaluating specific genotypes are warranted.

It is worth noting that only cefepime-resistant strains were selected to study the benefit of protecting cefepime by AAI101. Although this selection facilitated direct comparison of potency of various antibiotics, the reported susceptibilities do not represent those expected to be encountered routinely in the clinical setting. Although data for cefepime/AAI101 against a normal distribution are unavailable, consideration of cefepime alone against this population can provide valuable insights. Namely, against the full collection of >2500 strains from which the studied isolates were selected, the percentages of *E. coli* and *K. pneumoniae* isolates susceptible to cefepime were 85% and 87%, respectively [10]. Similarly, global data from the 2009 to 2012 SENTRY database reported cefepime percent susceptibility rates of 78%–90% for respiratory *E. coli* and *K. pneumoniae* collected from the USA, Europe, and the Mediterranean [11]. Taken collectively, the isolates studied herein conservatively represent the upper 22% of the *E. coli* and *K. pneumoniae* MIC distribution that would likely be encountered during daily clinical practice and consequently underestimate the activity of cefepime/AAI101 against the total population.

## 4. Experimental Section

Non-urine *Enterobacteriaceae* (118 *E. coli* and 105 *K. pneumoniae*) were selected from the Center for Anti-Infective Research and Development’s (Hartford, CT) isolate inventory, which contains organisms collected from 43 US hospitals during 2013–2014 [10]. Isolates were selected to capture the upper end of the MIC distribution for cefepime, focusing on those outside the susceptibility range (*i.e.*, >2 mg/L).

MICs were determined using broth microdilution as described by the Clinical and Laboratory Standards Institute (CLSI) [5]. Standard powders of cefepime, ceftazidime, ciprofloxacin, ertapenem, meropenem, piperacillin/tazobactam, and tobramycin were obtained from Sigma-Aldrich (St Louis, MO, USA), and AAI101 was provided by Allecra Therapeutics SAS (St-Louis, France). For cefepime/AAI101, doubling dilutions of cefepime were utilized in combination with a fixed concentration of 8 mg/L of AAI101 [12].

Isolates were characterized using current CLSI susceptibility breakpoints as follows: cefepime, MIC ≤ 2 mg/L; ceftazidime, MIC ≤ 4 mg/L; ciprofloxacin, MIC ≤ 1 mg/L; ertapenem, MIC ≤ 0.5 mg/L; meropenem, MIC ≤ 1 mg/L; piperacillin/tazobactam, MIC ≤ 16 mg/L; tobramycin, MIC ≤ 4 mg/L [5]. Multidrug resistance (MDR) was defined as resistance to 3 or more classes of antimicrobials (*i.e.*, carbapenems, cephalosporins, monobactams, aminoglycosides, penicillins or fluoroquinolones). All isolates were evaluated phenotypically for production of ESBLs using methods described by the CLSI [5]. Briefly, ceftazidime and cefotaxime MICs were determined with and without clavulanate; those isolates that exhibited MIC shifts of ≥8-fold in the presence of clavulanate were classified as ESBL producers. Isolates non-susceptible to meropenem (≥4 mg/L), regardless of results of ESBL evaluations, were examined for carbapenemase production using the CarbaNP test [13].

## 5. Conclusions

The addition of AAI101 restores the activity of cefepime against a highly resistant population of *E. coli* and *K. pneumoniae*. In an era of increasing resistance and limited therapeutic options, cefepime/AAI101 represents a new potential treatment option warranting continued development for difficult-to-treat Gram-negative pathogens.
